# Multifactorial Chylomicronemia Syndrome With a Rare APOA5 Variant and Recurrent Acute Pancreatitis: A Case Report From Rural Colombia

**DOI:** 10.7759/cureus.110889

**Published:** 2026-06-15

**Authors:** Carlos J Clavijo Rios, Juan S Bolivar Egurrola, Julian M Moreno Leon

**Affiliations:** 1 School of Medicine, Universidad del Rosario, Bogota, COL; 2 Department of Internal Medicine, Fundación Cardioinfantil - La Cardio, Bogota, COL

**Keywords:** apoa5, chylomicronemia syndrome, colombia, hypertriglyceridemia genetics, pcsk9 inhibitor, recurrent pancreatitis, severe hypertriglyceridemia, vus

## Abstract

Severe hypertriglyceridemia (triglycerides ≥ 500 mg/dL) carries a high risk of recurrent acute pancreatitis and cardiovascular disease. Pathogenic variants in the APOA5 gene are associated with hyperlipoproteinemia type V and familial hypertriglyceridemia; however, detailed case reports involving variants of uncertain significance (VUS) with a severe clinical phenotype and documented therapeutic response are rarely published. A 46-year-old woman from rural Supía, Caldas, Colombia, presented with persistent very severe hypertriglyceridemia (peak: 2,170 mg/dL), three hospitalizations for acute pancreatitis, and inadequate response to statins and fibrates. Targeted exome sequencing identified a heterozygous APOA5 VUS: c.694T>C, p.Ser232Pro (NM_052968.5), classified as PM2/PP3 per ACMG criteria (ClinVar: VCV002617654.3). An off-label PCSK9 inhibitor regimen achieved a greater than 90% triglyceride reduction (nadir: 118 mg/dL), though adherence has been intermittent due to economic and healthcare access barriers. This case highlights the diagnostic value of targeted genetic testing in severe familial hypertriglyceridemia and illustrates how an APOA5 VUS may underlie a severe multifactorial chylomicronemia phenotype. Segregation analysis and functional studies in first-degree relatives are warranted to support variant reclassification. This report also underscores the impact of healthcare access barriers on rare metabolic disease outcomes in rural Latin America.

## Introduction

Hypertriglyceridemia is classified according to the 2026 American College of Cardiology/American Heart Association (ACC/AHA)/Multisociety Guideline on the Management of Dyslipidemia into three clinically relevant tiers: elevated (175-499 mg/dL), primarily associated with increased atherosclerotic cardiovascular disease (ASCVD) risk; severe (500-999 mg/dL), at which lipoprotein lipase (LPL) capacity becomes saturated leading to chylomicronemia and substantially elevated pancreatitis risk; and very severe (≥ 1,000 mg/dL), at which pancreatitis prevention becomes the dominant therapeutic priority [1]. The etiology is frequently multifactorial, combining primary (monogenic or polygenic) and secondary causes, such as uncontrolled diabetes mellitus, hypothyroidism, obesity, or medications [2].

The APOA5 gene encodes apolipoprotein A-V, a key regulator of plasma triglyceride metabolism. Apolipoprotein A-V facilitates the LPL-mediated hydrolysis of triglycerides in chylomicrons and very-low-density lipoproteins (VLDL). Pathogenic variants in APOA5 have been associated with familial hyperlipoproteinemia type V (OMIM 144650) and familial hypertriglyceridemia (OMIM 145750), both inherited in an autosomal dominant pattern [3,4]. However, variants of uncertain significance (VUS) in this gene - particularly missense substitutions - present a diagnostic challenge, as their functional impact on triglyceride metabolism is not always established.

The variant c.694T>C, p.Ser232Pro in APOA5 has been previously identified in a Colombian screening program for familial chylomicronemia syndrome conducted in Pereira, Colombia (2010-2020). In that study, seven patients carried variants in either the APOA5 gene (c.694T>C, p.Ser232Pro) or the GPIHBP1 gene (c.523G>C, p.Gly175Arg) among 2,415 patients with severe hypertriglyceridemia over a decade, without further delineation of the number of carriers per variant [5]. No detailed longitudinal clinical characterization or therapeutic outcomes were reported for individual carriers.

Here, we present the first detailed case report of a patient carrying the APOA5 c.694T>C VUS with a very severe phenotypic presentation, recurrent acute pancreatitis, and a documented response to off-label PCSK9 inhibitor therapy, prepared following the CAse REport (CARE) guidelines [6].

## Case presentation

Patient information

A 46-year-old woman (date of birth: August 9, 1979) residing in the rural municipality of Supía, Caldas, Colombia, was referred to Endocrinology from primary care in her municipality for evaluation of severe refractory hypertriglyceridemia with suspected genetic etiology. She was affiliated with a public health insurance provider (EPS, Entidad Promotora de Salud, the Colombian mandatory health insurance system) and reported recurrent difficulties in obtaining prescribed medications secondary to dispensing failures by her insurer.

Medical history

Relevant personal medical history included mixed dyslipidemia, type 2 diabetes mellitus (T2DM) under follow-up, and multiple hospitalizations for acute pancreatitis attributed to severe hypertriglyceridemia over the preceding five years. Prior pharmacological management included statins and fibrates, with unsatisfactory clinical response and documented adherence difficulties.

Surgical history: Cholecystectomy at age 18 secondary to cholelithiasis, likely reflecting early-onset biliary complications in the context of chronic hypertriglyceridemia. Total thyroidectomy with lateral neck lymph node dissection and parathyroidectomy (date not available in records), resulting in permanent hypothyroidism and hypoparathyroidism requiring lifelong replacement therapy. Bilateral tubal ligation.

Current medications: Levothyroxine (thyroid hormone replacement); calcium carbonate and calcitriol (post-parathyroidectomy replacement); basal-bolus insulin regimen (insulin glargine and insulin aspart) for glycemic control; fenofibrate; omega-3 fatty acids; evolocumab (PCSK9 inhibitor, off-label). Adherence has been intermittent due to recurrent systematic dispensing failures: the patient's EPS repeatedly failed to fulfill payment obligations to contracted pharmacies, resulting in pharmacy refusal to dispense medications - including fenofibrate, omega-3 fatty acids, and evolocumab - despite valid prescriptions. These failures were not isolated events but a recurring pattern throughout the follow-up period.

Family history: The patient's mother had a history of dyslipidemia; no further characterization was available. The patient has children; no further details were available in the records. No first-degree relatives had been evaluated for lipid disorders at the time of this report.

Social history: Rural residence with limited access to specialist care and persistent barriers in medication dispensation through the public health system.

Clinical findings

At the time of Endocrinology evaluation, her weight was 74.4 kg, height was 1.52 m, BMI was 32.2 kg/m² (grade I obesity), and blood pressure was 128/66 mmHg. Obesity constitutes an independent secondary contributor to hypertriglyceridemia, compounding the underlying genetic predisposition. No cutaneous xanthomas, eruptive xanthomatosis, or lipemia retinalis were reported across the follow-up period.

Timeline

**Figure 1:**
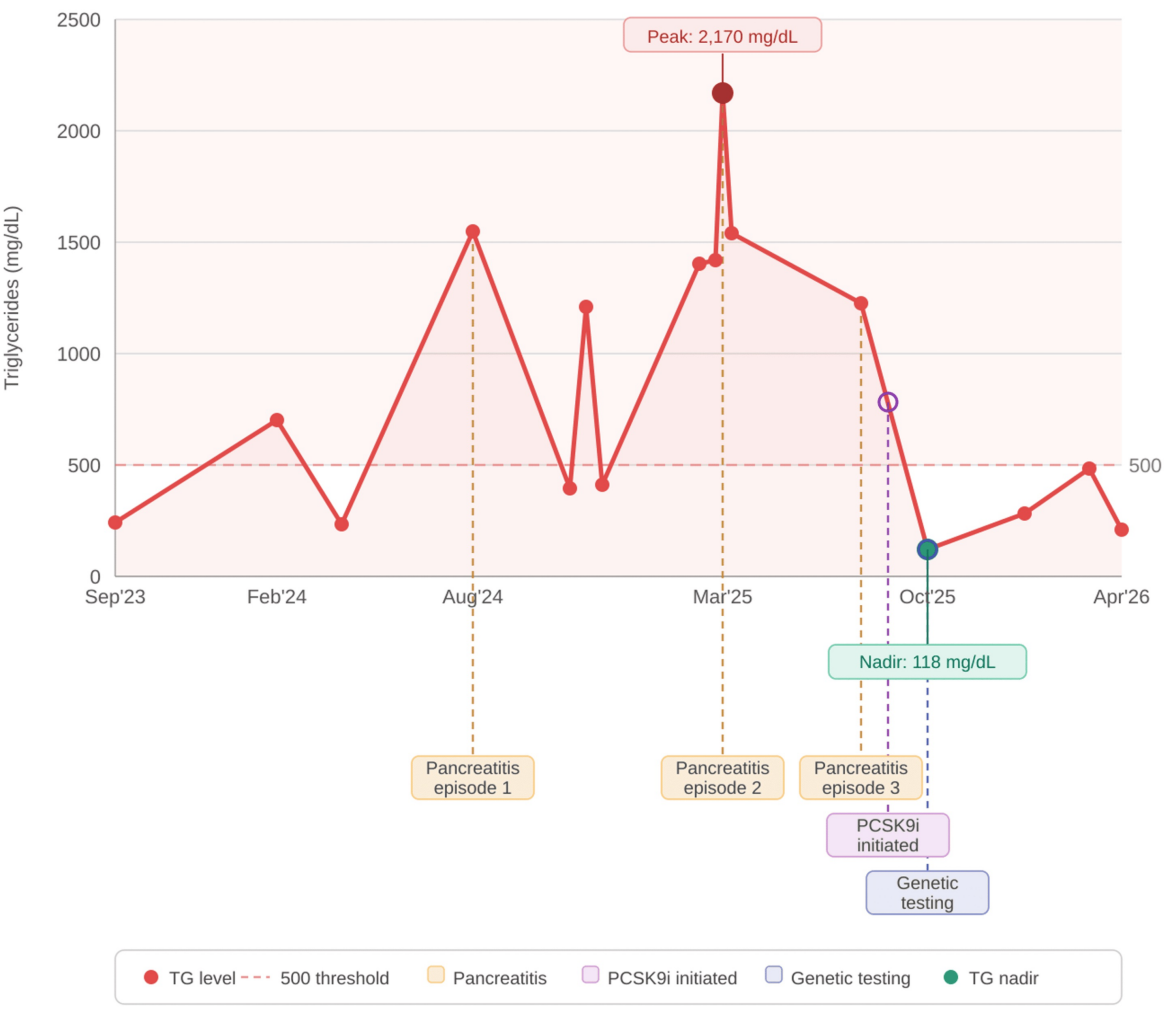
Triglyceride evolution and clinical events (2023-2026) Red line: TG level (mg/dL). Dashed red line: 500 mg/dL threshold for severe hypertriglyceridemia. Orange markers: acute pancreatitis episodes. Purple marker: PCSK9 inhibitor initiation (August 2025). Blue marker: genetic testing result reported (October 2025). Green dot: TG nadir (118 mg/dL). TG: triglycerides; PCSK9i: proprotein convertase subtilisin/kexin type 9 inhibitor.

Figure 1 illustrates the temporal evolution of triglyceride levels from September 2023 to April 2026. Multiple measurements exceeded 1,000 mg/dL, with a peak of 2,170 mg/dL in March 2025. A nadir of 118 mg/dL was recorded in October 2025 following initiation of the proprotein convertase subtilisin/kexin type 9 (PCSK9) inhibitor-based regimen, with subsequent triglyceride rebound upon treatment interruption.

Diagnostic assessment

Laboratory investigations: Table 1 summarizes the longitudinal lipid profile, HbA1c, and selected metabolic parameters from September 2023 to April 2026.

**Table 1:** Longitudinal lipid and metabolic profile (September 2023-April 2026) *TG ≥ 500 mg/dL (severe hypertriglyceridemia). †LDL not calculable due to TG > 400 mg/dL. —: not available. TG: triglycerides; TC: total cholesterol; HDL: high-density lipoprotein; LDL: low-density lipoprotein; HbA1c: glycated hemoglobin.

Date	TG (mg/dL)	TC (mg/dL)	HDL (mg/dL)	LDL (mg/dL)	HbA1c	Pancreatitis	Notes
Sep 2023	243	168	44	75	6.4%		TSH normal
Feb 2024	705*	205	30	—†	6.4%		TSH 2.57 mIU/L
Apr 2024	233	109	40	22	6.2%		
Aug 2024	1548*	490	24	—†	6.9%	Episode 1	Lipemic serum
Nov 2024a	393	166	54	33	9.3%		
Nov 2024b	1208*	303	31	—†	9.7%		Very lipemic serum
Dec 2024	410	204	33	—†	7.7%		TSH 1.59 mIU/L
Mar 2025a	1406*	602	22	—†	6.8%		Lipemic serum
Mar 2025b	1421*	727	18	—†	—		Very lipemic serum
Mar 2025c	2170*	—	—	—†	—	Episode 2	Peak TG - milky serum
Apr 2025	1539*	709	51	—†	—		Milky serum
Aug 2025	1225*	339	30	—†	7.0%	Episode 3	TSH 3.93; PCSK9i initiated
Oct 2025	118	154	49	81	9.0%		TG nadir; genetic testing reported
Jan 2026	286	170	53	60	12.0%		HbA1c peak
Mar 2026	480	181	50	—†	9.6%		
Apr 2026	209	201	55	104	7.9%		Most recent visit

Additional laboratory findings included TSH within therapeutic range on all three measurements (2.57, 1.59, and 3.93 mIU/mL), +consistent with adequate levothyroxine replacement; primary hypothyroidism as an independent contributor was thus excluded. Liver function tests (August 2025): aspartate aminotransferase (AST) 11 U/L, alanine transaminase (ALT) 13 U/L (normal), and total bilirubin 3.0 mg/dL. Renal function was preserved throughout (creatinine: 0.51-0.76 mg/dL). Complete blood counts and infectious serologies (HIV, HBsAg, syphilis) were negative.

Secondary causes of hypertriglyceridemia were systematically evaluated and excluded: hypothyroidism was excluded by serial thyroid-stimulating hormone (TSH) measurements within therapeutic range under levothyroxine replacement; renal and hepatic dysfunction were excluded by normal creatinine and transaminase levels throughout follow-up; excessive alcohol consumption was denied by the patient; and no lipid-elevating medications (corticosteroids, antipsychotics, antiretrovirals, or oral estrogens) were identified in the medication record.

Genetic testing: Targeted exome sequencing from peripheral blood was performed by Instituto de Diagnóstico Médico S.A. (IDIME) (ISO 9001 certified laboratory, Colombia; report date: October 16, 2025) for the condition of familial dyslipidemia. Table 2 summarizes the genetic findings.

**Table 2:** Genetic findings - targeted exome sequencing (APOA5) HGVS: Human Genome Variation Society nomenclature; AD: autosomal dominant; ACMG: American College of Medical Genetics and Genomics; VUS: variant of uncertain significance; PM2: absent/very low frequency in population databases; PP3: multiple lines of computational evidence supporting deleterious effect. ClinVar accession: VCV002617654.3

Gene	Transcript & Variant (HGVS)	Zygosity	Inheritance	Classification
APOA5	NM_052968.5: c.694T>C, p.Ser232Pro	Heterozygous	AD	VUS (PM2, PP3)

The identified variant, c.694T>C (p.Ser232Pro), results from a thymine-to-cytosine substitution at position 694 of the APOA5 coding sequence (exon 3/3), leading to a serine-to-proline amino acid change at position 232. The substitution may plausibly alter APOA5 protein conformation through incorporation of a structurally rigid proline residue, potentially impairing the ability of apolipoprotein A-V to function as a cofactor for LPL and reducing triglyceride hydrolysis [4]. The variant has an allele frequency of approximately 0.0003% in gnomAD with no reported homozygotes and is listed in ClinVar (VCV002617654.3) as a VUS per the American College of Medical Genetics and Genomics (ACMG)/AMP classification criteria (PM2: very low population frequency; PP3: computational evidence of deleterious effect) [7]. This variant was previously identified as a carrier finding in a Colombian cohort with severe hypertriglyceridemia, though without detailed clinical characterization [5]. No pathogenic copy number variants (CNVs) were identified by NGS. Given the autosomal dominant inheritance pattern and the presence of dyslipidemia in the patient's mother, genetic testing has been recommended for the mother and the patient's children; however, no first-degree relatives have undergone genetic evaluation to date.

Therapeutic interventions

Prior to the current evaluation, the patient had received sequential trials of statins and fenofibrate with inadequate triglyceride control and documented adherence difficulties secondary to recurrent dispensing failures by her public health insurer. In addition to pharmacological management, standard non-pharmacological measures were recommended, including a very low-fat diet, restriction of simple carbohydrates, avoidance of alcohol, and moderate aerobic physical activity. Following the endocrinology referral and genetic testing, the therapeutic regimen was restructured to address both the severe hypertriglyceridemia and poorly controlled diabetes mellitus.

Current pharmacological management includes (1) basal-bolus insulin therapy, indicated both for glycemic control in the context of T2DM and for a possible pancreatogenic (type 3c) diabetes component [8]; (2) fenofibrate for triglyceride-lowering; (3) omega-3 fatty acids as adjunctive lipid-lowering therapy; and (4) evolocumab, a PCSK9 inhibitor, initiated as an off-label adjunctive strategy for refractory mixed dyslipidemia in a genetically susceptible patient.

Rationale for PCSK9 inhibitor use: In the context of a likely APOA5-related hyperlipoproteinemia with mixed dyslipidemia - including episodes of markedly elevated total cholesterol (490 mg/dL, August 2024) and severe hypertriglyceridemia - and given the complete unavailability of olezarsen in Colombia due to its prohibitive cost and lack of regulatory reimbursement, evolocumab was prescribed as the most accessible advanced lipid-lowering option. Evidence supports an adjunctive role for PCSK9 inhibition in reducing triglyceride-rich remnant particles beyond LDL lowering, particularly in patients refractory to statins and fibrates [9]. It is important to note that PCSK9 inhibitors are not standard of care for hypertriglyceridemia and were used strictly off-label in this case, given the refractory nature of the dyslipidemia and the complete unavailability of olezarsen in Colombia. The theoretical rationale for their use in this context is as follows: PCSK9 inhibitors upregulate hepatic LDL receptors, enhancing clearance of apolipoprotein B-containing lipoproteins, including VLDL remnants and IDL particles. In the setting of impaired LPL-mediated triglyceride hydrolysis - as expected in APOA5-related dysfunction - this hepatic receptor-mediated clearance pathway may provide an adjunctive triglyceride-lowering effect. Evolocumab was well tolerated throughout the treatment period, with no injection site reactions, myalgias, or other adverse effects reported.

Following intensification of the multimodal regimen, triglyceride levels decreased to a nadir of 118 mg/dL in October 2025, representing a reduction of more than 90% from peak values of 2,170 mg/dL. Given the simultaneous optimization of multiple agents, the individual contribution of evolocumab cannot be isolated; however, the magnitude of response is notable and consistent with an adjunctive lipid-lowering effect. Adherence has been intermittent due to economic constraints and recurrent medication dispensing failures, resulting in subsequent triglyceride rebound.

Follow-up and outcomes

The patient experienced three hospitalizations for acute pancreatitis between 2023 and 2025, all managed conservatively without ICU admission or surgical intervention, resolving without documented complications. All three episodes were diagnosed according to the revised Atlanta Classification criteria, requiring at least two of the following: characteristic abdominal pain, serum lipase or amylase greater than three times the upper limit of normal, and/or confirmatory cross-sectional imaging. Specific laboratory values and imaging reports were not retrievable from available records.

Regarding diabetes, recurrent episodes of acute pancreatitis and likely underlying chronic pancreatic inflammation may have further impaired endocrine pancreatic function over time, contributing to progressive deterioration in glycemic control (HbA1c ranging from 6.2% in April 2024 to 12.0% in January 2026). This pattern raises suspicion for a pancreatogenic component (type 3c) superimposed on pre-existing insulin resistance, and warrants formal evaluation of exocrine pancreatic function.

At the most recent visit (April 2026), triglycerides measured 209 mg/dL with HbA1c of 7.9%, reflecting partial but incomplete metabolic control. The patient remains under active endocrinology follow-up. Genetic testing of the patient's mother and children has been formally recommended, though logistic and economic barriers have thus far prevented its realization. Formal assessment of exocrine pancreatic function, including fecal elastase measurement and dedicated pancreatic imaging, was not available in the records and represents an important limitation. Detailed glycemic monitoring beyond HbA1c - including fasting glucose, C-peptide levels, and insulin dose titration records - was similarly unavailable. Regarding functional outcomes, no formal quality of life assessment was conducted; however, no further pancreatitis hospitalizations have been recorded following initiation of the current multimodal regimen, representing a clinically meaningful reduction in disease burden compared to the preceding two-year period.

## Discussion

This case presents a 46-year-old Colombian woman with very severe hypertriglyceridemia, recurrent acute pancreatitis, and a heterozygous APOA5 VUS (c.694T>C, p.Ser232Pro). The clinical phenotype - triglyceride levels repeatedly exceeding 1,000 mg/dL, low HDL-cholesterol, lipemic serum, and three pancreatitis episodes - is compatible with a multifactorial chylomicronemia/type V phenotype. The p.Ser232Pro substitution may plausibly alter APOA5 protein conformation through incorporation of a structurally rigid proline residue, potentially impairing LPL interaction - supported by computational evidence (PP3) and very low population frequency (PM2) [4,7]. A possible pancreatogenic contribution (type 3c) is clinically relevant [8]; however, the persistence of triglycerides above 500 mg/dL even during reasonable glycemic control supports a primary genetic substrate. Primary hypothyroidism was excluded by TSH measurements within therapeutic range under levothyroxine replacement. Preserved hepatic and renal function confirms the absence of end-organ damage.

An important conceptual distinction merits discussion. Classic monogenic familial chylomicronemia syndrome (FCS) results from biallelic pathogenic variants in LPL, APOC2, APOA5, LMF1, or GPIHBP1 and is characterized by extreme fasting hypertriglyceridemia largely unresponsive to conventional therapy [10]. Multifactorial chylomicronemia syndrome (MCS), by contrast, results from heterozygous susceptibility variants - such as a heterozygous APOA5 variant - combined with secondary amplifying factors including obesity, insulin resistance, and uncontrolled diabetes. The incomplete penetrance and variable expressivity characteristic of heterozygous APOA5 variants may explain why severe phenotypic expression often requires these secondary metabolic stressors as 'second hits'. This patient's profile - heterozygous APOA5 VUS, grade I obesity, T2DM with variable glycemic control, and partial response to multimodal therapy - is more consistent with MCS than classic FCS. This distinction matters clinically: MCS patients may derive greater benefit from optimizing secondary factors, while FCS patients typically require RNA-based therapies such as olezarsen. Formal lipoprotein characterization, including apolipoprotein B quantification and electrophoresis, was unavailable in the treating setting, limiting definitive phenotypic classification [2,3].

Following intensification of multimodal lipid-lowering and glycemic therapy - including basal-bolus insulin, fenofibrate, omega-3 fatty acids, and off-label evolocumab - triglyceride levels decreased to a nadir of 118 mg/dL, representing a reduction of more than 90% from peak values. Given the simultaneous optimization of multiple agents, the individual contribution of evolocumab cannot be isolated; however, the magnitude of response suggests a possible adjunctive role in refractory mixed dyslipidemia with a genetic susceptibility component [9]. Evolocumab was chosen as the most accessible strategy given the complete unavailability of olezarsen (the approved therapy for familial chylomicronemia [11]) in Colombia. Evidence supports an adjunctive role for PCSK9 inhibition in reducing triglyceride-rich remnant particles beyond LDL lowering, particularly in patients refractory to statins and fibrates [9,12]. The triglyceride rebound upon treatment interruption underscores the need for uninterrupted therapy to prevent pancreatitis recurrence.

From a healthcare equity perspective, this case illustrates the compounded barriers faced by patients with rare genetic metabolic disorders in rural Colombia: recurrent medication dispensing failures, limited specialist access, and economic constraints that interrupt potentially life-saving therapy. These structural failures may have contributed to the recurrent episodes of severe hypertriglyceridemia and pancreatitis. Hypertriglyceridemia-induced acute pancreatitis is associated with higher mortality, increased pancreatic necrosis, and greater intensive care requirements than other etiologies [13] - risks that are amplified when the underlying genetic cause remains undiagnosed.

The autosomal dominant inheritance pattern extends the clinical urgency beyond the index patient. The patient's mother - who has documented dyslipidemia (and her children - each with a 50% probability of carrying the variant) - should undergo lipid profiling and, if feasible, genetic testing. Co-segregation of the variant with the phenotype in affected relatives would contribute supportive evidence toward reclassification, as per ACMG/AMP criteria. In rural communities with limited genetic services, such variants may go unrecognized across generations, perpetuating preventable morbidity and mortality.

Comparison with previously reported APOA5 cases is informative. Published case reports have predominantly described homozygous or compound heterozygous APOA5 variants causing classic FCS phenotypes, with triglyceride levels frequently exceeding 20-30 mmol/L and largely refractory to conventional therapy. Makhmudova et al. reported a homozygous APOA5 frameshift variant (c.427delC, p.Arg143Alafs57) in a 25-year-old patient with triglycerides up to 29.8 mmol/L and recurrent pancreatitis requiring volanesorsen [14]. The present case is distinctive in several respects: the variant is heterozygous and classified as a VUS; the phenotype is consistent with MCS rather than classic FCS; secondary metabolic factors, including obesity and T2DM, significantly amplify the phenotypic expression; and the therapeutic response was achieved with off-label PCSK9 inhibition in a resource-limited setting where approved therapies were unavailable. To our knowledge, no previously published case has described this specific combination of features.

The main limitations include the following: absence of exocrine pancreatic function testing, lack of formal lipoprotein characterization (ApoB, electrophoresis), no family genetic testing, VUS classification of the variant, and intermittent treatment adherence. Future case series and prospective documentation of PCSK9 inhibitor response in genetically confirmed hypertriglyceridemia would strengthen the evidence base.

## Conclusions

We report a case of very severe hypertriglyceridemia with three episodes of acute pancreatitis in a 46-year-old rural Colombian woman carrying a heterozygous APOA5 VUS (c.694T>C, p.Ser232Pro, NM_052968.5), whose clinical profile is most consistent with multifactorial chylomicronemia syndrome (MCS) amplified by T2DM, obesity, and a possible pancreatogenic contribution. Off-label PCSK9 inhibitor therapy - as part of a multimodal regimen and in the absence of olezarsen - was associated with a greater than 90% triglyceride reduction, suggesting a possible adjunctive role in genetically susceptible mixed dyslipidemia. Given the autosomal dominant inheritance, the index case may represent the visible tip of an unrecognized burden of disease in her rural community. This case reinforces the value of genetic testing in severe familial dyslipidemia, highlights the importance of distinguishing FCS from MCS, and underscores the urgent need to extend genetic screening and specialist care to vulnerable rural populations in Latin America. Prospective documentation of PCSK9 inhibitor response in heterozygous APOA5 carriers, combined with functional studies and segregation analysis in first-degree relatives, may inform future therapeutic guidelines and contribute to reclassification of this variant from VUS to likely pathogenic.
